# Automated Speech Analysis for Risk Detection of Depression, Anxiety, Insomnia, and Fatigue: Algorithm Development and Validation Study

**DOI:** 10.2196/58572

**Published:** 2024-10-31

**Authors:** Rachid Riad, Martin Denais, Marc de Gennes, Adrien Lesage, Vincent Oustric, Xuan Nga Cao, Stéphane Mouchabac, Alexis Bourla

**Affiliations:** 1 Callyope Paris France; 2 Department of Psychiatry, Saint-Antoine Hospital Sorbonne University Assistance publique - Hôpitaux de Paris Paris France; 3 Infrastructure for Clinical Research in Neurosciences Paris Brain Institute Paris France; 4 Medical Strategy and Innovation Department Clariane Paris France; 5 NeuroStim Psychiatry Practice Paris France

**Keywords:** speech analysis, voice detection, voice analysis, speech biomarkers, speech-based systems, computer-aided diagnosis, mental health symptom detection, machine learning, mental health, fatigue, anxiety, depression

## Abstract

**Background:**

While speech analysis holds promise for mental health assessment, research often focuses on single symptoms, despite symptom co-occurrences and interactions. In addition, predictive models in mental health do not properly assess the limitations of speech-based systems, such as uncertainty, or fairness for a safe clinical deployment.

**Objective:**

We investigated the predictive potential of mobile-collected speech data for detecting and estimating depression, anxiety, fatigue, and insomnia, focusing on other factors than mere accuracy, in the general population.

**Methods:**

We included 865 healthy adults and recorded their answers regarding their perceived mental and sleep states. We asked how they felt and if they had slept well lately. Clinically validated questionnaires measuring depression, anxiety, insomnia, and fatigue severity were also used. We developed a novel speech and machine learning pipeline involving voice activity detection, feature extraction, and model training. We automatically modeled speech with pretrained deep learning models that were pretrained on a large, open, and free database, and we selected the best one on the validation set. Based on the best speech modeling approach, clinical threshold detection, individual score prediction, model uncertainty estimation, and performance fairness across demographics (age, sex, and education) were evaluated. We used a train-validation-test split for all evaluations: to develop our models, select the best ones, and assess the generalizability of held-out data.

**Results:**

The best model was Whisper M with a max pooling and oversampling method. Our methods achieved good detection performance for all symptoms, depression (Patient Health Questionnaire-9: area under the curve [AUC]=0.76; *F*_1_-score=0.49 and Beck Depression Inventory: AUC=0.78; *F*_1_-score=0.65), anxiety (Generalized Anxiety Disorder 7-item scale: AUC=0.77; *F*_1_-score=0.50), insomnia (Athens Insomnia Scale: AUC=0.73; *F*_1_-score=0.62), and fatigue (Multidimensional Fatigue Inventory total score: AUC=0.68; *F*_1_-score=0.88). The system performed well when it needed to abstain from making predictions, as demonstrated by low abstention rates in depression detection with the Beck Depression Inventory and fatigue, with risk-coverage AUCs below 0.4. Individual symptom scores were accurately predicted (correlations were all significant with Pearson strengths between 0.31 and 0.49). Fairness analysis revealed that models were consistent for sex (average disparity ratio [DR] 0.86, SD 0.13), to a lesser extent for education level (average DR 0.47, SD 0.30), and worse for age groups (average DR 0.33, SD 0.30).

**Conclusions:**

This study demonstrates the potential of speech-based systems for multifaceted mental health assessment in the general population, not only for detecting clinical thresholds but also for estimating their severity. Addressing fairness and incorporating uncertainty estimation with selective classification are key contributions that can enhance the clinical utility and responsible implementation of such systems.

## Introduction

Depression and anxiety disorders are recognized as the leading causes of disease burden [1], and their prevalences are high during the entire life span across the sexes and all around the globe [2]. This burden was aggravated by the COVID-19 pandemic [3]. In these disorders, early identification and evaluation of the severity of the symptoms are of prime importance since the incidence of suicide is associated with a diagnosis of depression more than 50% of the time [4]. Besides, measurement-based care, via the use of clinically valid scales, improves the follow-up and treatment of affected individuals with mental health disorders [5]. Timely interventions lead to better outcomes in mental health. This proactive approach can ensure early access to treatment and prevent significant complications. Yet, measuring mental health remains a challenge, since manifestations of depression and anxiety are heterogeneous [6] and co-occur with insomnia [7] and fatigue [8]. The exhaustive and objective assessment of these different mental health dimensions through validated assessment scales is long and fastidious for clinical staff and is particularly not adapted to primary care, which is at the forefront of handling mental health disorders [9]. The development of objective biomarkers, which are easy to collect without the synchronization of clinicians and patients, has the potential to overcome these limitations. These quantifiable measures, encompassing biological, genetic, or behavioral assessments, could revolutionize early detection, enabling timely and targeted interventions that ultimately improve the patient’s outcomes and well-being. This is particularly significant for screening the general population across diverse mental health dimensions. Indeed, screening the first signs of mental health problems or symptoms could help to avoid escalation of symptoms, as different dimensions interact in time [7,10].

The study of speech biomarkers in mental health holds great potential, offering a noninvasive and easily accessible avenue to capture significant motor, cognitive, and behavioral changes due to mental health disorders such as depression [11-14]. Clinical evidence and research studies have increasingly linked specific automated extracted speech features, such as prosody, articulation, and fluency, with various mental health conditions, such as depression [11,15], anxiety [16], suicide-risk assessment [17], fatigue [18,19], or sleep deprivation [20]. The complexity of human speech extends beyond the intricate motor coordination involved. The speech production system within the brain relies on the synchronization of diverse cognitive, social, and motor processes [21,22]. This intricate interplay involves hundreds of muscles across the respiratory, phonatory, and supralaryngeal systems, working in concert with critical cognitive skills like attention, memory, and planning. Additionally, social skills such as theory of mind and emotional processing play a vital role. Importantly, disruptions in any of the aforementioned motor, cognitive, or social skills, as well as mental health states, can introduce perturbations in the resulting speech signal. Besides, beyond research evidence, clinical practitioners also use voice unconsciously when evaluating individuals, and these subjective evaluations could be complemented and refined with objective measures from automatic speech analysis.

Speech biomarkers emerge also as a promising avenue for mental health assessment due to their unique characteristics: they are noninvasive, cost-effective, and convenient tools. Recent hardware and software advancements have significantly simplified and reduced the cost of acquiring acoustic data, making it a more accessible option compared to traditional biological, imaging, or cognitive markers. In addition, speech data collection requires minimal effort from both patients and clinicians and can even be conducted remotely, further enhancing its feasibility in various settings.

However, despite its promises, the study of speech biomarkers remains largely fragmented, in laboratory settings, or not evaluated for deployment into clinical practice. This gap calls for more evidence to be integrated into clinical practice [23]. Research on speech in mental health in the general population often focuses on 1 isolated mental health dimension, even though there are proofs supporting the existence of networks of symptoms and syndromes in mental health that influence each other [24,25]. In addition, previous speech studies were limited to specific populations such as students [26] or older people [27]. However, before machine learning (ML) models can be used in clinical settings to make predictions for individuals, they must be “fair”—providing equally accurate predictions across all demographic groups [28]. Finally, speech-based systems should not be tested only for the simple classification of binary labels (eg, depressed or not depressed) but rather for the estimation of the severity of symptoms [29], and their ability to refrain from giving an output when uncertainty is too high, therefore deferring decisions to the health staff in practice [30].

In this study, the main objective was to assess the predictive potential of speech models in detecting and estimating the severity of depression, anxiety, fatigue, and insomnia within the general population using mobile-collected speech and mental health data. Besides, to prove that these models could be effectively implemented in diverse real-world settings, they are assessed for their fairness and uncertainty capabilities.

## Methods

### Participants

We recruited French healthy adult participants without any known severe psychiatric or neurological disorder (self-declaration) or speech pathologies such as stuttering or clutter.

### Ethical Considerations

All participants signed an informed consent form to participate in the study, in line with the Declaration of Helsinki, current Good Clinical Practice guidelines, and local laws and regulations. All procedures were approved by the French National Institutional Review Board (identifier 23.00748.OOO2L7#I for the Committee for the Protection of Persons). All data were stored on secure health data servers, without any identifying information in the metadata. Participants received a €15 gift card as compensation for their time.

### Study Procedure

#### Overview

**Figure 1:**
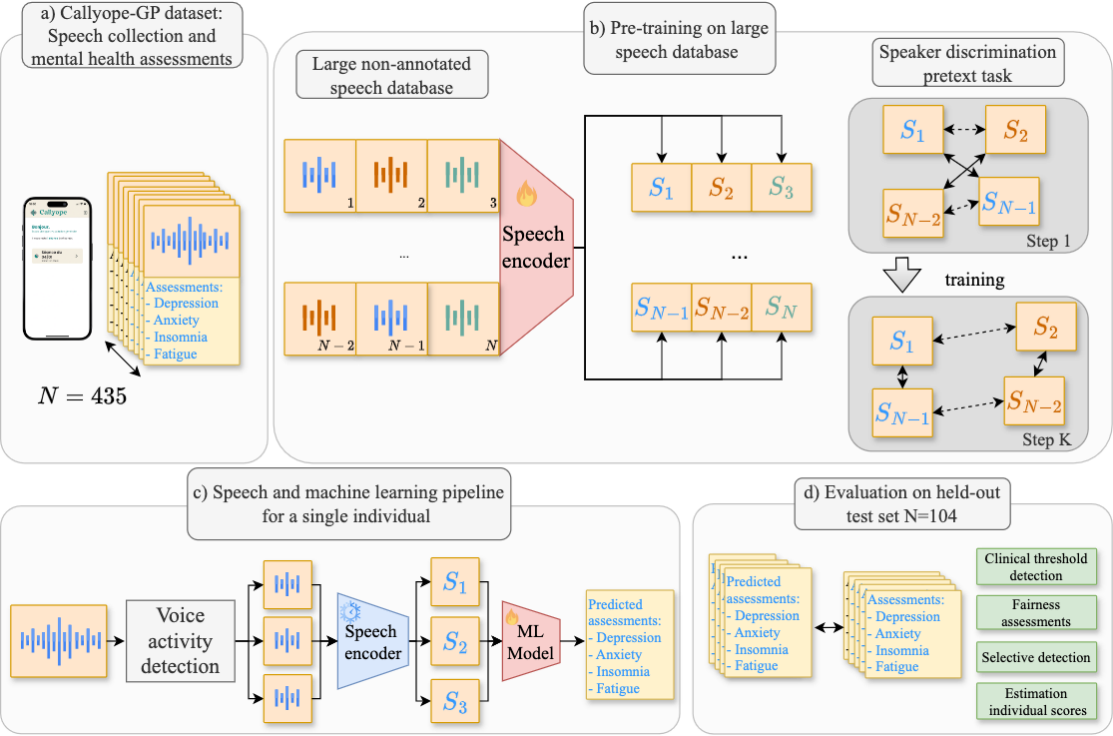
Overview of this study. (A) Overview of our Callyope General-Population (Callyope-GP) dataset with 865 included participants. (B) Flowchart of the pretraining phases of our speech encoders with an illustration of the pretraining speaker embedding process. (C) Graphical illustration of our speech and machine learning (ML) pipeline for a single individual. The pretrained speaker embedding is frozen, and only ML models on top are trained on training data. (D) Evaluation and comparison between true and predicted assessment on held-out participants (test set n=131). Si represents the speech turn vector embeddings obtained from the speech encoder model, colors represent speaker identities for each speech turn and embedding, and wi represents the words spoken in the given audio.

The participants completed the protocol on smartphones through the Callyope research mobile app in a home environment. The participants completed self-assessment scales for different mental health dimensions and recorded different speech tasks. In this work, we only focused on 1 spontaneous and semistructured speech task where participants had to answer “Describe how you are feeling at the moment and how your nights’ sleep have been lately” [31]. The participants were included by speech pathologist interns and recruited through social media platforms. Finally, self-reported symptoms were examined with clinically validated questionnaires (Figure 1A). Participants followed the instructions displayed on the Callyope app, and their vocal answers were recorded with the smartphone’s microphone. The audio was sampled at 44.1 kHz with a 16-bit resolution. Each participant was asked to place his phone on a flat surface (eg, a table) with the microphone pointing toward the speaker, that is, himself. The session should take place in a quiet environment, whenever possible.

We refer to the dataset collected in this study as the Callyope General-Population (Callyope-GP) dataset. We split randomly the Callyope-GP dataset into 3 sets: training, validation, and testing. Demographic data, such as sex, age, and education level, were collected. We compared groups with adequate tests for their demographics and self-assessments to ensure that groups were consistent.

#### Measures of Depressive Symptoms

To allow broader use of our solutions, the severity of depression was assessed through the Beck Depression Inventory (BDI) [32] and Patient Health Questionnaire-9 (PHQ-9) [33] self-report questionnaires. In current clinical practices, it is common that different professionals interacting with a patient use different metrics to monitor depression. While depression assessment through these 2 measures exhibits a robust correlation at the group level [34], thus facilitating the development of an equational conversion for research uses, their limited efficacy at the individual level impedes their reliable conversions to predict individual depressive status [35].

The PHQ-9 is a short, self-administered questionnaire mainly used to screen and measure the severity of depression [33] and is sensitive to potential changes [34]. It includes the 2 cardinal signs of depression: anhedonia and depressed mood. We considered a risk of depression if the total score for the PHQ-9 was more than 10 (PHQ-9≥10).

The BDI is a self-administered questionnaire with 21 items, each centered around a core theme [32,36]. Respondents are presented with statements for each item, and they are instructed to choose 1 statement, which is then associated with a score ranging from 0 to 3. The cumulative score for the scale can reach a maximum of 63 points. We considered the BDI threshold to be positive if the total score was more than 10 (BDI≥10), as it is above the normal range as defined by the authors of the BDI [36].

#### Measure of Anxiety

The Generalized Anxiety Disorder 7-item scale (GAD-7) questionnaire is to measure or assess the severity of GAD [37]. This is a self-administered questionnaire that takes less than 5 minutes to complete, and it was especially developed to be deployed efficiently in primary care. The optimal cutoff for the GAD-7 was found to be a cutoff for the total score of GAD-7≥10 [37].

#### Measure of Insomnia

The Athens Insomnia Scale (AIS) is a self-administered questionnaire to assess the patient’s sleep difficulties according to the *International Classification of Diseases, Tenth Revision* (ICD-10) criteria [38]. The AIS-8 comprises 8 items (5 minutes) and is a good tool for general sleep assessment and insomnia screening and to measure the intensity of sleep-related problems but also as a screening tool in reliably establishing the diagnosis of insomnia. The optimal cutoff for diagnosis to detect insomnia troubles, for the AIS scale, is 6 [39,40].

#### Measures of Fatigue

We used the Multidimensional Fatigue Inventory (MFI) to assess the different dimensions of fatigue [41-43]. It is a short self-report questionnaire (5-10 minutes) based on 20 questions to determine 5 dimensions of fatigue: general fatigue, physical fatigue, reduced motivation, reduced activity, and mental fatigue. We also reported the total fatigue score as the sum of all subcomponents.

We used the normative data from Schwarz et al [42] and Hinz et al [44] to choose thresholds for each subcomponent. Individual subcomponents of fatigue in the 75% quantile in the studied populations are all above 10. Therefore, we aimed to predict individuals’ scores, which are above or equal to 10, for each dimension. As mentioned also in Schwarz et al [42], the total score has clinical significance and validity, as it was observed to have the highest correlations with anxiety, depression, and quality of life. There is no consensus cutoff for the total sum fatigue score; yet, based on the Colombian normative data [44], we observed that the mean values for each studied subgroup were all above 40; therefore, we chose a clinical threshold of 40 for the total sum score.

### ML Analyses

#### Overview

**Figure 2:**
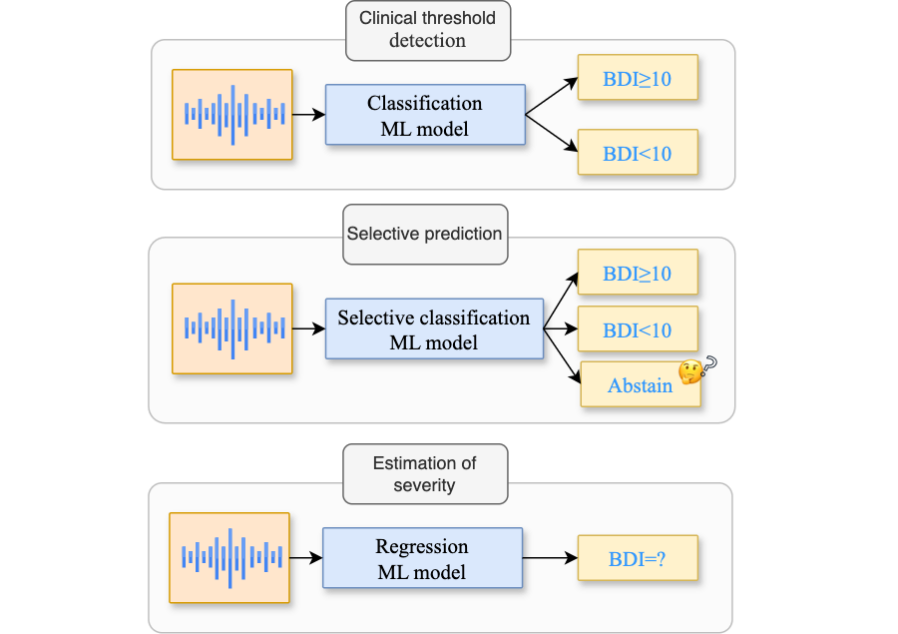
Schematic representation of the clinical tasks that are being assessed in this study. Each task is different in terms of the set and types of outputs. The different tasks were illustrated with the BDI clinical scale and its given threshold of 10. BDI: Beck Depression Inventory; ML: machine learning.

**Figure 3:**
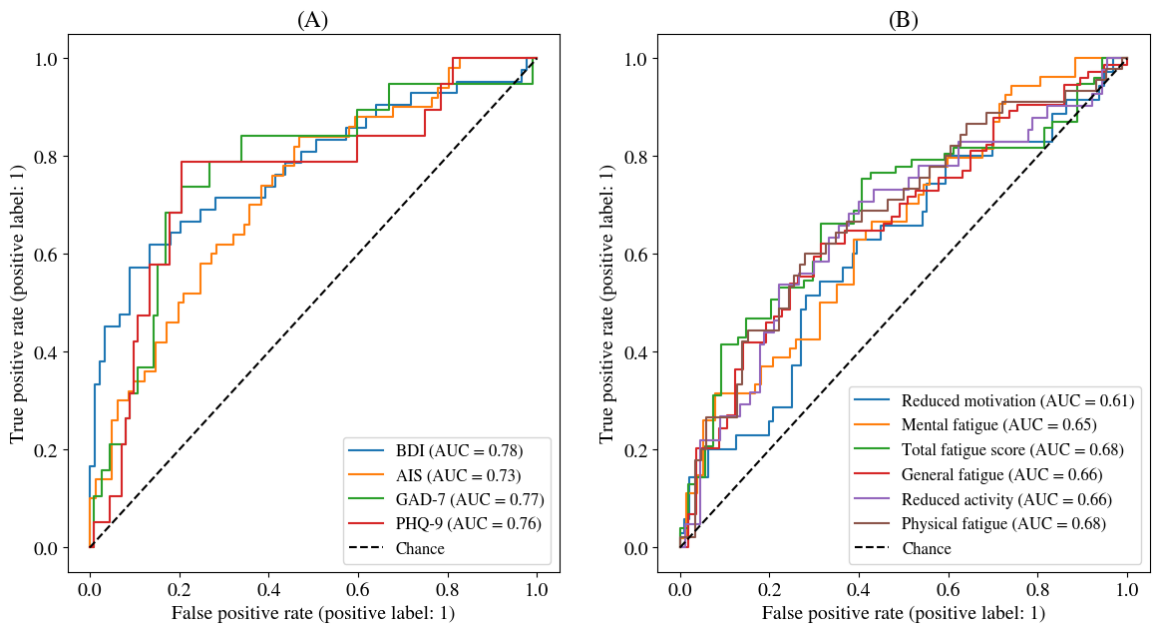
ROC curves for the clinical threshold detection task on the held-out test set. (A) ROC curves to detect clinically relevant thresholds for depression (PHQ-9 and BDI), anxiety (GAD-7), and insomnia (AIS). (B) ROC curves to detect clinically relevant thresholds for fatigue components (MFI), general fatigue, physical fatigue, reduced activity, mental fatigue, reduced motivation, and total fatigue. AUC: area under the curve; AIS: Athens Insomnia Scale; BDI: Beck Depression Inventory; GAD-7: General Anxiety Disorder 7-item scale; MFI: Multidimensional Fatigue Inventory; PHQ-9: Patient Health Questionnaire-9; ROC: receiver operating characteristic curve.

Our ML analyses can be decomposed into three main steps: (1) the pretraining of the speech encoder model (Figure 1B) (2) the fine-tuning of ML models for each mental health aspect considered in this study (Figure 1C), and (3) extensive evaluations of the clinical threshold detection, selective detection, fairness assessments, and severity estimations for each clinical scale (Figures 1D and 2).

#### Model Pretraining and Tuning

Audio intensity is normalized per sample, and we compared the three main approaches to obtain representations for large-scale speech models: (1) Speaker recognition is performed using a ThinResNet model with 34 layers. The model takes speech samples as input, which are encoded as 40-Mel spectrograms, with a hop length of 10 ms and a Hamming window. This architecture is based on the ResNet design introduced by He et al [45]. (2) We also considered a transformer [46] architecture adapted for speech (HuBERT [47]), trained in a self-supervision fashion, that is, to predict masked neighbor embeddings. (3) Finally, we evaluated a transformer architecture Whisper pretrained to tackle automatic speech recognition.

Speaker recognition as a pretraining task has proven great results in mental health and neurology [12,48]. The ThinResNetis pretrained on the VoxCeleb2 dataset [49], which is publicly available and contains over 1 million utterances from 6112 speakers, from 145 nationalities. The VoxCeleb2 dataset consists of almost only continuous speech. The pretraining learning forces the model to organize speech in terms of speaker characteristics, as we illustrated earlier in the right panel of Figure 1B. We used an additive-margin softmax loss for this speaker identification task [50].

We also compared these models to a self-supervised model, HuBERTXL, which exhibits great generalization for paralinguistic tasks such as emotion recognition [51]. HuBERT was trained on 960 hours of Librispeech [52]. Whisper is a recent robust automatic speech recognition system based on a transformer architecture trained on 680,000 hours of transcribed speech. Whisper training data are much bigger and more diverse with noisy labels than other speech models. We considered 3 versions of the model: small, medium, and large.

In this work, we did not fine-tune any of the speech encoder models on the Callyope-GP dataset, which we represented in Figure 1C by a frozen speech encoder model. For each speaker *i*, we obtained a vector representation, a speech vector embedding denoted *S_i_* We extracted segments of 20 seconds with 10 seconds overlap, and we compared different pooling of predictions with mean and max pooling. Besides, as our data are imbalanced, we also compared classic sampling of examples to train models with undersampling of majority class and oversampling of minority classes. The default values of undersampling and oversampling were used. We found no differences with and without voice activity detection, so we used windowing for simplicity. Extraction was performed using Python (version 3.9; Python Software Foundation), and the following packages were used to extract the acoustic features: *pytorch 2.0.1*, *imbalanced learn*, *torchaudio 2.0.2*, and *voxceleb_trainer project* [53].

For the fine-tuning of each task and each clinical score, different ML algorithms were compared on the validation set. For each task, once a model was selected, we retrained this final model on the concatenation of the training and validation sets and tested on the held-out test to avoid any inflated results. We used the scikit-learn implementation of each algorithm, splitting and evaluation [54].

For the speech collected in the Callyope-GP dataset, we applied the frozen speech encoder to each speech turn and propagated mental health assessment labels at the speech turn level to train and compare the final ML model. At inference, for final evaluation, we pool predictions at the speaker level, after we obtained varying speech turns from a specific speaker. We illustrated in Figure 2 each clinical end point, translated as an ML task based on the aforementioned procedure.

#### Clinical Threshold Detection (Classification)

We first compared the predictive power of the speech encoder to discriminate between individuals who are below or above the threshold for each clinical scale. The distributions of positive and negative labels vary across clinical dimensions, and to take into account imbalance, we reported the performances of the macro *F*_1_-scores along the area under the curve (AUC) of the receiver operating characteristic on the test set.

We compared linear-based models (logistic regression for classification with L2 regularization and elastic net linear model for regression), tree-based models (random forests with 100 estimators), and gradient-boosting algorithms (histogram-based gradient boosting). Even though the pretraining phase captured information about the participants’ mental health, it is important to build a final model for each mental health dimension to be more specific and more sensitive. In ML terms, the mental health characteristics in speech are not necessarily linearly separable in the last vector space of the speech encoder.

#### Estimation of Severity Through Predictions of Individual Scores (Regression)

The conventional approach in mental health assessment through speech analysis typically focuses on the group’s statistical analyses or binary classifications of categorical outcomes, primarily discerning the presence or absence of a specific dimension. Yet, the risks of depression, anxiety, fatigue, and insomnia exist along a spectrum of severity levels that exert varying degrees of influence on an individual’s well-being.

We go beyond traditional prediction and categorizations by integrating the estimation of severity through predictions of individual scores using regression ML models. This offers a more nuanced and comprehensive understanding of mental health dynamics, allowing for a more refined and personalized assessment. We evaluated our estimation of the severity of each total score with the mean absolute error (MAE) between actual and predicted scores and Pearson correlations. MAE score directly measures how close the predicted scores are to the actual scores without considering the direction of error. We also reported the Pearson correlation and the *P* value between the actual test set and the predicted values.

#### Fairness Assessments: Quality of Services for Sex, Age, and Education Level Demographics

ML systems can behave unfairly for different reasons and in multiple ways [28]. In medicine, the use of ML and predictive models should be carefully evaluated, especially for potential quality-of-service harms, that is, it can occur when a system is not as performant for 1 specific group of people as it is for another group. We conducted a thorough analysis of the quality of service regarding potential harms for each clinical scale in the final predicted model across every dimension: sex, age, and education level. The disparity ratio (DR) was reported based on clinical threshold detection *F*_1_-scores [55] to consider both false positives and false negatives and to be more stringent than equality of opportunity. The DR is computed as the fraction of the minimum *F*_1_-score across subgroups divided by the average *F*_1_-score on the full test set:

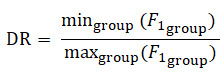



We did the same for Pearson correlation for regression. The higher the DR, the better it is, as it means that the model performs equally well across groups with the perfect DR being 1, that is, each group has the same level of performance. The *fairlearn* toolkit was used to perform our fairness evaluations [56].

#### Selective Clinical Threshold Detection (Selective Prediction)

ML approaches have made great strides in several domains; yet, apps to high-stakes settings remain challenging. In our case, in mental health assessments, communicating appropriately the uncertainty associated with the system predictions is critical [57]. Yet, the communication of probabilities to human users is hard [58], and a pragmatic approach is to determine if an artificial intelligence system is more likely to make erroneous predictions and defer these cases to clinicians. This approach can be viewed as a selective prediction task, where the ML system has the ability to withhold a prediction when it is too uncertain (essentially, the model saying “I don’t know”) [59,60]. In this work, we followed the method from Hendrycks and Gimpel [61], and we used the maximum output probabilities of the ML classification system as a way to measure uncertainty.

Based on a moving threshold, we can obtain a specific ML system to choose to abstain when its output probabilities are too low. This specific ML system is evaluated based on the predictions it chooses to make only; thus, there is a specific coverage and a specific accuracy or risk. There is a natural tradeoff between the coverage of the ML system and its accuracy. Therefore, the way to evaluate a selective prediction task is the AUC for the risk-coverage curve.

## Results

### Data Overview and Demographics of Participants

A total of 1150 participants were eligible and agreed to participate in our study. Among them, 865 completed the study, giving a recruitment yield of 75.2%. There was an equal split between Android (n=475, 54.9%) and iOS (n=390, 45.1%) devices used by participants. The reasons for which participants were not included in this analysis were the following: one missing speech task, missing demographic information, or one missing answer in the self-report questionnaires.

For our analyses, 605 participants were in the training set, 129 were in the validation set, and 131 were in the test set, and these groups did not differ in terms of demographics and mental health evaluations (Table 1). This yields a dataset sufficient (n>500) to evaluate error bars and predictive algorithms to avoid over-optimistic results [62]. Among the 865 participants, 275 (31.8%) were above the BDI screening threshold, 146 (16.9%) were above the PHQ-9 threshold, 133 (15.3%) were above the GAD-7 threshold, 371 (42.5%) were above the AIS threshold, 489 (56.5%) were above the MFI general fatigue threshold, 325 (37.5%) were above the MFI physical fatigue threshold, 283 (32.7%) were above the MFI reduced activity threshold, 379 (43.8%) were above the MFI mental fatigue threshold, 209 (24.1%) were above the MFI reduced motivation threshold, and 557 (64.4%) were above the threshold of the total score of the MFI. We reported a co-occurrence matrix of people at risk for each dimension in Figure S1 in Multimedia Appendix 1.

**Table 1:** Demographic characteristics of the training, validation, and test groups^a^.

Characteristics^b^				Training (n=605)	Validation (n=129)		Test (n=131)	Group comparisons				
								*F* test (*df*)			*P* value	
**Demographics**												
	**Sex, n (%)**								9.0^c^ (4)			.06
		Female	386 (76.4)		85 (65.9)	69 (52.2)						
		Male	214 (35.3)		44 (34.1)	63 (47.7)						
		Other	5 (0.8)		0 (0)	0 (0)						
	**Education level, n (%)**								5.2^c^ (6)			.52
		No diploma	19 (3.1)		7 (5.4)	8 (6.1)						
		Secondary studies	107 (17.7)		21 (16.2)	24 (18.3)						
		Short postbaccalaureate studies (Bac+2)	71 (11.7)		20 (15.5)	16 (12.2)						
		Long postbaccalaureate studies (Bac+3 and above)	408 (67.4)		81 (62.8)	83 (63.3)						
	**Age (years)**								0.78 (2)			.45
		Mean (SD)	38.8 (18.2)		40.2 (18.7)	40.1 (20.0)						
		Range	18-92		18-86	18-89						
**Clinical evaluation**												
	**BDI^d^**								0.07 (2)			.92
		Mean (SD)	7.7 (7.3)		7.9 (7.2)	7.9 (7.7)						
		Range	0.0-32.0		0.0-29.0	0.0-36.0						
		Negative, n (%)	413 (68.3)		88 (68.2)	89 (67.9)						
		Positive^e^, n (%)	192 (31.7)		41 (31.8)	42 (32.1)						
	**PHQ-9^f^**								0.85 (2)			.42
		Mean (SD)	5.6 (4.5)		5.2 (4.7)	5.1 (4.4)						
		Range	0.0-23.0		0.0-25.0	0.0-22.0						
		Negative, n (%)	499 (82.5)		108 (83.7)	112 (85.5)						
		Positive^e^, n (%)	106 (17.5)		21 (16.3)	19 (14.5)						
	**GAD-7^g^**								0.50 (2)			.60
		Mean (SD)	5.0 (4.5)		4.8 (4.7)	5.4 (5.0)						
		Range	0.0-21.0		0.0-20.0	0.0-21.0						
		Negative, n (%)	511 (84.5)		109 (84.5)	112 (85.5)						
		Positive^e^, n (%)	94 (15.5)		20 (15.5)	19 (14.5)						
	**AIS^h^**								1.6 (2)			.19
		Mean (SD)	5.6 (3.9)		5.0 (3.1)	5.2 (4.1)						
		Range	0.0-24.0		0.0-16.0	0.0-19.0						
		Negative, n (%)	338 (55.9)		75 (58.1)	81 (61.8)						
		Positive^e^, n (%)	267 (44.1)		54 (41.8)	50 (38.2)						
	**MFI^i^ general fatigue**								1.8 (2)			.17
		Mean (SD)	10.4 (4.2)		11.1 (4.2)	10.4 (4.1)						
		Range	4.0-20.0		4.0-20.0	4.0-20.0						
		Negative, n (%)	335 (55.4)		80 (62)	74 (56.5)						
		Positive^e^, n (%)	270 (44.6)		49 (38)	57 (43.5)						
	**MFI physical fatigue**								0.55 (2)			0.57
		Mean (SD)	8.7 (3.9)		8.8 (4.0)	8.4 (3.9)						
		Range	4.0-20.0		4.0-20.0	4.0-20.0						
		Negative, n (%)	376 (62.1)		78 (60.5)	86 (65.6)						
		Positive^e^, n (%)	229 (37.9)		51 (39.5)	45 (34.4)						
	**MFI reduced activity**								0.92 (2)			.40
		Mean (SD)	8.3 (3.8)		7.9 (3.5)	8.1 (3.6)						
		Range	3.0-20.0		4.0-20.0	4.0-18.0						
		Negative, n (%)	400 (66.1)		92 (71.3)	90 (68.7)						
		Positive^e^, n (%)	205 (33.9)		37 (28.7)	41 (31.3)						
	**MFI mental fatigue**								0.26 (2)			.77
		Mean (SD)	9.2 (4.2)		9.5 (4.4)	9.3 (4.2)						
		Range	4.0-20.0		4.0-20.0	4.0-20.0						
		Negative, n (%)	340 (56.2)		69 (53.5)	77 (58.8)						
		Positive^e^, n (%)	265 (43.8)		60 (46.5)	54 (41.2)						
	**MFI reduced motivation**								0.74 (2)			.47
		Mean (SD)	7.5 (3.0)		7.2 (3.2)	7.7 (3.2)						
		Range	4.0-20.0		4.0-20.0	4.0-18.0						
		Negative, n (%)	458 (75.7)		102 (79.1)	96 (73.3)						
		Positive^e^, n (%)	147 (24.3)		27 (20.9)	35 (26.7)						
	**MFI total score**								0.09 (2)			.91
		Mean (SD)	44.2 (15.1)		44.6 (15.0)	43.9 (15.1)						
		Range	19.0-99.0		20.0-90.0	20.0-83.0						
		Negative, n (%)	338 (55.9)		79 (61.2)	77 (58.8)						
		Positive^e^, n (%)	267 (44.1)		50 (38.8)	54 (41.2)						

^a^Categorical variables are compared with Pearson chi-square test, and continuous variables are compared with 1-way ANOVA.

^b^Characteristics for the 3 splits of the dataset to ensure generalization.

^c^Chi-square test.

^d^BDI: Beck Depression Inventory.

^e^In addition to statistics for each clinical scale score, we report the participants below and above the cutoff (see the *Methods* section for each threshold).

^f^PHQ-9: Patient Health Questionnaire-9.

^g^GAD-7: General Anxiety Disorder 7-item scale.

^h^AIS: Athens Insomnia Scale.

^i^MFI: Multidimensional Fatigue Inventory.

### ML Analyses

#### Overview

**Figure 4:**
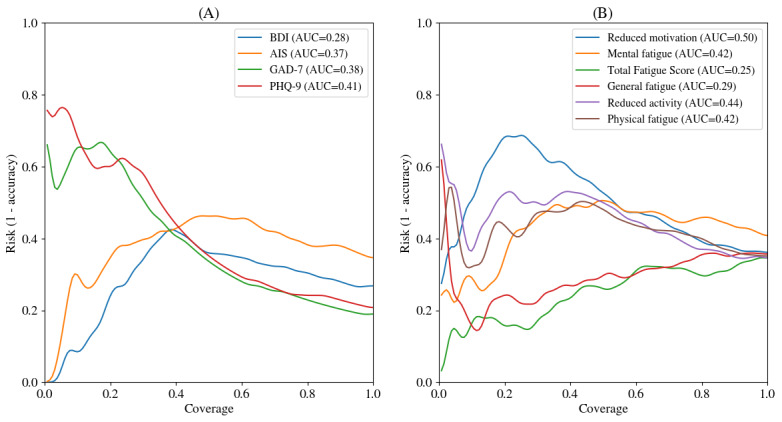
Risk-coverage curves for the selective clinical threshold detection task on the held-out test set illustrate the models’ selective screening ability, that is, risk detection capabilities with the ability to abstain when too uncertain. Curves are smoothed for clarity with a Gaussian blur but not used to compute AUC. A lower AUC is better, and 0 is the perfect score. (A) Risk-coverage curves selectively detect clinically relevant thresholds for depression (PHQ-9 and BDI), anxiety (GAD-7), and insomnia (AIS). (B) Risk-coverage curves to detect clinically relevant thresholds for fatigue components (MFI), general fatigue, physical fatigue, reduced activity, mental fatigue, reduced motivation, and total fatigue. AIS: Athens Insomnia Scale; AUC: area under the curve; BDI: Beck Depression Inventory; GAD-7: General Anxiety Disorder 7-item scale, MFI: Multidimensional Fatigue Inventory; PHQ-9: Patient Health Questionnaire-9.

Models and pipelines were compared with the results on the validation set (Table S1 in Multimedia Appendix 1). Overall, all the Whisper models outperformed other approaches (Whisper M being the best with mean *F*_1_-score=0.56, SD 0.09), while the speaker model performed the worst, even though still performing well. We found out that the performance of the pooling with the maximum prediction always outperformed the mean pooling, and undersampling and oversampling were helping. It was found out that the linear-based models were outperforming random forest and gradient boosting on the validation set. Thus, we retrained linear algorithms with max pooling and the Whisper M frozen speech encoder on the combination of the training and validation sets and reported the final results on the held-out test set in Figures 3 and 4 and Tables 2-4.

**Table 2:** Estimation of clinical threshold detection severity results on the held-out test set for the different considered dimensions of mental health (classification).

Dimensions of mental health	*F*_1_-score^a^
PHQ-9^b^	0.49
GAD-7^c^	0.50
BDI^d^	0.65
AIS^e^	0.62
MFI^f^ general fatigue	0.66
MFI physical fatigue	0.54
MFI reduced activity	0.52
MFI mental fatigue	0.53
MFI reduced motivation	0.43
MFI total fatigue score	0.69

^a^Higher *F*_1_-score is better, and 1 is perfect.

^b^PHQ-9: Patient Health Questionnaire-9.

^c^GAD-7: General Anxiety Disorder 7-item scale.

^d^BDI: Beck Depression Inventory.

^e^AIS: Athens Insomnia Scale.

^f^MFI: Multidimensional Fatigue Inventory.

**Table 3:** Disparity ratios (DRs) based on the F1-scores for sex, age, and education levels to assess fairness on the held-out test set for the clinical threshold detection (classification) for the different considered dimensions of mental health^a^.

Dimensions of mental health	DR				Mean (SD)^b^
	Sex	Age^c^	Education level^d^		
PHQ-9^e^	0.61	0.00	0.00	0.20 (0.35)	
GAD-7^f^	0.66	0.00	0.00	0.22 (0.38)	
BDI^g^	0.85	0.25	0.57	0.56 (0.30)	
AIS^h^	0.98	0.45	0.69	0.71 (0.27)	
MFI^i^ general fatigue	0.77	0.73	0.75	0.75 (0.02)	
MFI physical fatigue	0.97	0.76	0.90	0.88 (0.11)	
MFI reduced activity	0.95	0.27	0.56	0.59 (0.34)	
MFI mental fatigue	0.91	0.00	0.58	0.50 (0.46)	
MFI reduced motivation	0.94	0.25	0.30	0.50 (0.38)	
MFI total fatigue score	0.91	0.62	0.35	0.63 (0.28)	
Mean (SD)^j^	0.86 (0.13)	0.33 (0.30)	0.47 (0.30)	0.55 (0.33)	

^a^Higher DR is better, and 1 is perfect DR.

^b^Mean and SD values per score.

^c^Participants are grouped into age categories to allow analysis: 18-30, 30-45, 45-65, and >65 years.

^d^Highest achieved study level: no diploma or secondary studies or short postbaccalaureate studies (Bac+2) or long postbaccalaureate studies (Bac+3 and above).

^e^PHQ-9: Patient Health Questionnaire-9.

^f^GAD-7: General Anxiety Disorder 7-item scale.

^g^BDI: Beck Depression Inventory.

^h^AIS: Athens Insomnia Scale.

^i^MFI: Multidimensional Fatigue Inventory.

^j^Mean and SD are reported per sensitive dimension.

**Table 4:** Estimation of severity results on the held-out test set for the different considered dimensions of mental health (regression)^a^.

Dimensions of mental health	Pearson *r*	MAE^b^
PHQ-9^c^	0.47	3.1
GAD-7^d^	0.48	3.2
BDI^e^	0.49	4.9
AIS^f^	0.43	2.9
MFI^g^ general fatigue	0.38	3.3
MFI physical fatigue	0.32	3.0
MFI reduced activity	0.31	2.9
MFI mental fatigue	0.34	3.1
MFI reduced motivation	0.32	2.5
MFI total fatigue score	0.44	11.3

^a^We reported mean absolute errors and Pearson correlations between actual and predicted values. Lower mean absolute error is better, and 0 is perfect. A higher Pearson correlation is better, and +1 is perfect. All correlations were significant (*P*<1×10^–14^).

^b^MAE: mean absolute error.

^c^PHQ-9: Patient Health Questionnaire-9.

^d^GAD-7: General Anxiety Disorder 7-item scale.

^e^BDI: Beck Depression Inventory.

^f^AIS: Athens Insomnia Scale.

^g^MFI: Multidimensional Fatigue Inventory.

#### Clinical Threshold Detection (Classification)

The clinical threshold detection performed well based on the speech data and our developed system (Figure 3 and Table 2). All systems outperformed the chance levels. Based on the AUC, the classification results were the highest for the BDI score (AUC=0.78; *F*_1_-score=0.65). Based on the *F*_1_-score, it was the total MFI score (AUC=0.68; *F*_1_-score=0.69). The lowest for both metrics was the MFI reduced motivation (AUC=0.61; *F*_1_-score=0.43).

#### Fairness Assessments: Quality of Services for Sex, Age, and Education Level for Classification

We computed the sensitive attribute DRs (sex, age, and education level) and assessed the differences in the quality of the service made by the speech-based system (Table 3) for classification. Overall, the classification of the speech-based system had a better quality of service for sex (mean 0.86, SD 0.13), and the worst was for age (mean 0.33, SD 0.30). We also identified that the detection of PHQ-9 had the worst quality-of-service disparity (mean of DRs 0.20, SD 0.35), and the best quality-of-service was obtained with the AIS (mean of DRs 0.71, SD 0.27), the MFI general fatigue (mean of DRs 0.75, SD 0.02), and the MFI physical fatigue (mean of DRs 0.88, SD 0.11). We also observed that only the MFI general fatigue and MFI physical fatigue obtained a good performance for age (MFI general fatigue: DR=0.73 and MFI physical fatigue: DR=0.76), and, except for PHQ-9 and GAD-7, all DRs were satisfactory or high for sex.

#### Selective Clinical Threshold Detection (Selective Prediction)

The capabilities of speech-based models were also evaluated to selectively predict the different clinical thresholds. Great performances were observed for BDI, AIS, and MFI general fatigue (Figure 4). The model that selectively predicts the risk of depression based on the BDI score achieved the best result (BDI risk-coverage: AUC=0.28). The other scores could not achieve such feats of important coverage with no risk.

#### Estimation of Severity Through Predictions of Individual Scores (Regression)

The regression results for the estimation of severity are reported in Table 3. Speech-based models obtained significant results for all clinical variables based on the evaluation with the Pearson correlations (all *P*<1×10^–14^). The strongest correlations between the prediction and the actual scores on the held-out test were found for BDI score (*r*=0.49), GAD-7 (*r*=0.48), and PHQ-9 (*r*=0.47). The lowest correlation was found for the MFI reduced activity (*r*=0.31).

Speech-based models also obtained great results in terms of absolute errors. We observed less than 3 points of MAE for AIS and MFI reduced activity. All other scores were predicted on average with less than 5 points, except for the MFI total fatigue score, since its range is 13-88.

## Discussion

### Principal Findings

We aimed to explore the full capabilities and limitations of using speech data extracted from 865 participants in the general population to predict the presence or absence of different mental health self-reported symptoms: depression, anxiety, and insomnia and the different dimensions of fatigue. We built a fully automated speech-based ML system that takes as input the audio waveform collected from 1 simple speech task performed on our smartphone app. The models were trained and calibrated on training and validation sets of participants, and we demonstrated the system’s generalization on the held-out test set of 131 participants.

The results indicated that ML-based systems using speech only as input could identify participants above clinical thresholds for depression, insomnia, total fatigue components, but to a lesser extent, anxiety and fatigue subcomponents. All classification results were above chance levels for each clinical threshold.

This result was confirmed with an extensive fairness analysis of quality of service for age, sex, and education levels. Depression, insomnia, and different dimensions of fatigue clinical threshold detection results were particularly consistent for sex, slightly less for age, and to a lesser extent for education level. Anxiety risk identification fell behind in accuracy overall and was also unequal per group. The extension of our clinical threshold detection system to be able to abstain, with selective prediction, was conclusive, even for anxiety. Risk-coverage AUCs remained low for insomnia, total fatigue, and depression detection through BDI. Finally, we showed that speech-based models could also predict the severity, with the prediction of exact scores moving beyond binary interpretations of score thresholds. All correlations between the predicted scores and the actual scores given by participants were significant, exhibiting strengths ranging from 0.31 to 0.49.

Our study builds upon existing mental health research on speech analysis and extends the insights for deployment into clinical practice. For risk and anxiety depression assessments from speech in the general population, we found similar strong performances such as in previous studies using speech analyses [26,27,63,64]. Our recruitment and involvement of participants was in person. For medium-sized datasets with face-to-face recruitment (below 1000 participants, such as ours), it can be observed that crowdsourced recruitment of participants via web-based platforms needs more training data to yield the same level of model accuracy [63,65]. This was also observed in a large study, with over 6000 participants, with web-based data recruitment for risk detection in the general population in American English [64]. This discrepancy could be explained by the fact that data quality is variable on web crowdsourcing platforms, especially for participants’ psychiatric evaluations [66], and also voice recording through laptops.

Our study revealed discrepancies in both clinical threshold detection and estimation of severity through self-reported depression scores between BDI and PHQ-9. This underlines the inherent limitations of score conversion and the crucial role of individual-level assessment in capturing the nuanced and different expressions of depression [35]. This reinforces the necessity of developing assessment tools and interpreting results with meticulous attention to individual variability, particularly by scrutinizing model performance at the individual level, mirroring real-world clinical scenarios.

Our study uniquely addresses the co-occurrence of perceived fatigue and reported insomnia, both prevalent mental health concerns, which could be detected simultaneously through speech analysis. While prior research, like the work by Thoret et al [20], has explored how sleep deprivation impacts specific vocal features like prosody and voice quality, no previous study has delved into the combined influence of fatigue and insomnia on speech. Addressing this gap is crucial because these conditions often co-occur and significantly impact the symptom trajectory and potential development of other mental health issues [67]. The prevention of recurrent sleep problems can prevent other mental health troubles or relapses. The observed co-occurrence of symptoms, particularly insomnia with other clinical dimensions, highlights the interconnected nature of these symptoms and syndromes.

Finally, to the best of our knowledge, this is the first study to assess the fairness and selective prediction capabilities in speech-based mental health assessments. This is of prime importance since speech signals can be heavily influenced by a multitude of factors such as age, sex, weight, and height [68]. Age factor was the least preserved in our grouped performances, this can be attributed to voice changes due to hormones [69], and normal aging affects the different parts of the vocal production system: larynx, respiratory system, resonators, saliva system, and the individual’s global emotional status [70]. In addition to lower performances compared to other mental health dimensions, the anxiety risk detection performance collapsed for certain groups of demographics. This could be explained by the heterogeneity and low positive examples in our Callyope-GP dataset. Even though there are limits concerning some groups of individuals, selective classification offers an option to potentially remediate these variable quality of services, ensuring a deployment in clinical settings and still bringing overall clinical utility.

### Limitations

While valuable, this study has some limitations. The French monolingual, medium-sized dataset (300<n<1000) needs more diverse data to achieve better generalizability, and the nonlongitudinal design misses insights on symptom evolution. Besides, the use of self-assessment scales introduces a potential bias because they rely heavily on the insight of participants. A limitation of our study is the use of a fixed train-dev-test split. This split approach, while convenient for comparing results across different tasks, can introduce bias and limit the generalizability of our findings. Future research with larger samples, longitudinal designs, and the inclusion of pathological data is crucial for exploiting the full potential of voice biomarkers in mental health. Studying various and diverse speech tasks and prompts also holds the potential for multiple benefits related to user adherence. Indeed, tailored tasks can address specific mental health needs, cater to individual preferences, and boost engagement. Varying prompts can reduce user fatigue and sustain interest, leading to more consistent system use and richer data collection.

### Conclusions

This study demonstrates the potential of speech-based systems for detecting and predicting various mental health symptoms in the general population. While challenges remain regarding real-world apps and ensuring fairness across the population demographics, our findings pave the way for further development and responsible integration of such tools into clinical settings, advancing personalized mental health assessment and intervention. In future work, we will extend this study by including longitudinal data, adding more diverse linguistic and geographic data, and including more severely affected patients who are already followed by mental health practitioners. We will also look into fairness and uncertainty mitigation methods to improve the performance of our systems.

## Appendix

Multimedia Appendix 1 Co-occurrences of symptoms, and validation and comparison results for each speech modeling approach.

## Abbreviations

AIS: Athens Insomnia Scale
AUC: area under the curve
BDI: Beck Depression Inventory
Callyope-GP: Callyope General-Population
DR: disparity ratio
GAD-7: General Anxiety Disorder 7-item scale
ICD-10: International Classification of Diseases, Tenth Revision
MAE: mean absolute error
MFI: Multidimensional Fatigue Inventory
ML: machine learning
PHQ-9: Patient Health Questionnaire-9
RCT: randomized controlled trial
