# Supplementary material for: Automated Speech Analysis for Risk Detection of Depression, Anxiety, Insomnia, and Fatigue: Algorithm Development and Validation Study
Source: J Med Internet Res. 2024 Oct 31;26:e58572. doi: 10.2196/58572 (PMC11565087; doi:10.2196/58572)
Supplement: Multimedia Appendix 1 [file jmir_v26i1e58572_app1.docx]

**Figure S1.** Co-occurrences percentage of people at risk for each dimension, normalization is done per row: first row can be read as 57% of individuals who are at risk for depression based on the PHQ-9 are at risk of GAD-7.
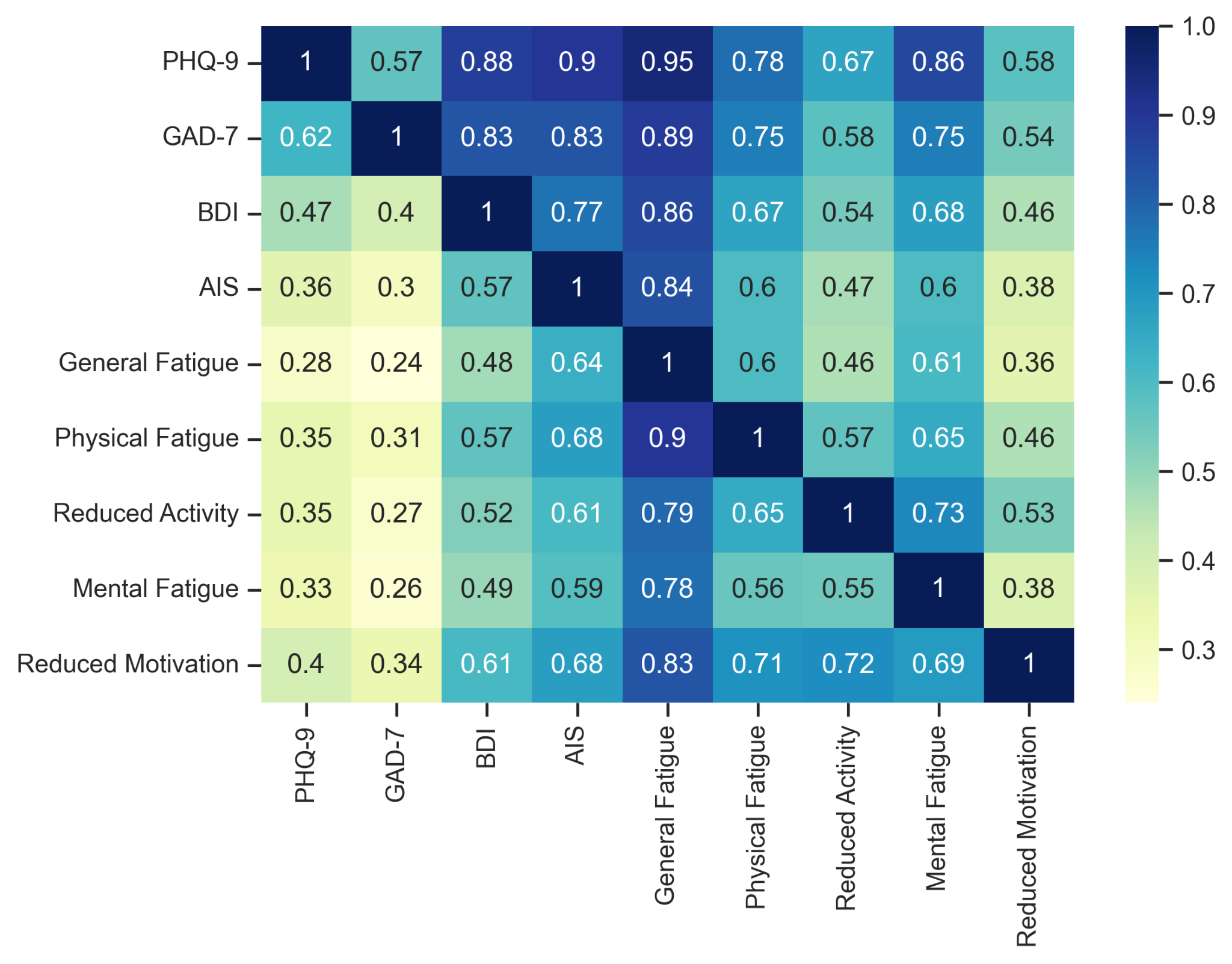


**Table S1.** Results on the validation set for the clinical threshold detection task on the validation set for the F1 score and AUC score, averaged across clinical scales. We displayed the top 3 approach for each model. We underlined the best approach per frozen speech model, and bolded the best overall.

| Frozen speech model | Pooling | Sampling | Classifier | F1 score |
| --- | --- | --- | --- | --- |
| Random classifier (Dummy) | n/a | n/a | n/a | 0.15 |
| Speaker Recognition ResNetSE34 | max | undersample | Logistic Regression | 0.518 |
|  | max | undersample | Random Forest | 0.514 |
|  | max | oversample | Logistic Regression | 0.510 |
| Self-supervised  HubertXL | max | oversample | Logistic Regression | 0.532 |
|  | max | undersample | Logistic Regression | 0.528 |
|  | max | undersample | Gradient boosting | 0.522 |
| WhisperS | max | oversample | Logistic Regression | 0.535 |
|  | max | undersample | Logistic Regression | 0.522 |
|  | max | None | Logistic Regression | 0.513 |
| WhisperM | max | oversample | Logistic Regression | 0.560 |
|  | max | oversample | Gradient boosting | 0.547 |
|  | max | undersample | Logistic Regression | 0.542 |
| WhisperL | max | oversample | Logistic Regression | 0.548 |
|  | max | undersample | Logistic Regression | 0.545 |
|  | max | undersample | Gradient boosting | 0.527 |
